# Real-Time Fluorometric Isothermal LAMP Assay for Detection of *Chlamydia pecorum* in Rapidly Processed Ovine Abortion Samples: A Veterinary Practitioner’s Perspective

**DOI:** 10.3390/pathogens10091157

**Published:** 2021-09-08

**Authors:** Tom Clune, Susan Anstey, Vasilli Kasimov, Caroline Jacobson, Martina Jelocnik

**Affiliations:** 1 Centre for Animal Production and Health, Murdoch University, South Street, Murdoch, Perth, WA 6150, Australia; c.jacobson@murdoch.edu.au; 2 Genecology Research Centre, University of the Sunshine Coast, Sippy Downs, Sunshine Coast, QLD 4557, Australia; susan.anstey@research.usc.edu.au (S.A.); vasilli.kasimov@research.usc.edu.au (V.K.); mjelocni@usc.edu.au (M.J.)

**Keywords:** *Chlamydia pecorum*, loop-mediated isothermal amplification, sheep, point-of-care, abortion

## Abstract

Traditional methods of detecting *Chlamydia pecorum* in tissue samples such as polymerase chain reaction or cell culture are laborious and costly. We evaluated the use of a previously developed *C. pecorum* LAMP assay using minimally processed ovine samples. Cotyledon (*n* = 16), foetal liver (*n* = 22), foetal lung (*n* = 2), and vaginal (*n* = 6) swabs, in addition to cotyledon (*n* = 6) and foetal liver (*n* = 8) tissue samples, were rapidly processed and used for LAMP testing without DNA extraction. Overall, LAMP test results were highly congruent with the in-house reference qPCR, with 80.43% (37/46; 72.73% positive agreement (PA); 84.75% negative agreement (NA)) overall agreeance for swab samples, and 85.71% (12/14; 80% PA; 88.89% NA) overall agreeance for tissue samples. Out of the 11 total discrepant results, discrepancy was mainly observed in samples (*n* = 10) with less than 100 copies/µL *C. pecorum* DNA. While sensitivity could be improved, the simplicity, low cost, and accuracy of detection makes this test amenable for use at point-of-care for detecting *C. pecorum* in sheep.

## 1. Introduction

*Chlamydia pecorum*, an obligate intracellular bacterium and a member of *Chlamydiacae*, is a significant global livestock and marsupial pathogen. In sheep, *C. pecorum* is a common cause of polyarthritis and conjunctivitis [1,2,3,4,5]. However, this organism is also frequently detected in the faeces of apparently healthy sheep [6,7]. Recently, *C. pecorum* has been implicated in cases of ovine abortion across Australia, with diagnosis aided by bacterial isolation and/or nucleic acid amplification assays performed in specialised diagnostic laboratories [8,9]. While these methods are considered the ‘gold-standard’ for detecting and diagnosing *C. pecorum*, they are laborious, time-consuming, and costly [10]. 

An alternate method of pathogen detection by loop-mediated isothermal amplification (LAMP) is becoming popular, with numerous published assays for various bacterial, protozoal, and viral veterinary pathogens [11]. Recently, Jelocnik et al. [12] developed a rapid isothermal testing assay for *C. psittaci* and *C. pecorum* and successfully demonstrated its use with DNA extracts from samples taken from a range of animal hosts (sheep, cattle, koalas, and horses). The initial study also described a rapid swab processing method, utilising vortexing to dislodge cells from clinical swabs, followed by heat lysis to release DNA to decrease sample processing time and use of commercial DNA extraction kits. Another isothermal *C. pecorum* assay for detection of koala *C. pecorum* infections was recently proposed by Hulse et al. [13], who used KOH to lyse cells and a specific isothermal mastermix (Lyse N LAMP mix, Optigene, UK). However, the limitation of this assay is that, upon testing, the sample is no longer viable for DNA extraction and molecular characterisation of the infecting strains (where the sample tests positive for *C. pecorum*). Nevertheless, the utility of these assays at clinical setting and point-of-care (POC) is evident in koala clinical practice, where several wildlife hospitals utilise the *C. pecorum* LAMP assays as the diagnostic toolkit [12,13] (personal communication Dr Amy Robbins, Endeavour Veterinary Ecology, and Dr Amber Gillet, Australia Zoo Wildlife Hospital). The utility of chlamydial rapid isothermal testing and rapid sample processing has also been demonstrated in equine clinical practice to detect *C. psittaci* in equine abortions, both at POC and in the diagnostic laboratories [14]. 

Considering the emerging evidence for the abortigenic potential of *C. pecorum* infections in sheep [8,9,15], a rapid diagnosis would offer effective on-farm surveillance, improve biosecurity, and aid in infection control [14]. While the rapid swab processing and isothermal testing has been evaluated for koala *C. pecorum* detection, there is a paucity of reports for the use of these in diagnosing *C. pecorum* infections in sheep. In this pilot study, we evaluated the *C. pecorum* LAMP assay [12] for testing rapidly processed sheep swab and tissue samples collected during field investigations for abortion and stillbirth cases, and compared the LAMP assay to an in-house *C. pecorum* qPCR assay. 

## 2. Results

### 2.1. Limit of Detection of the C. pecorum LAMP Assay

This study used a *C. pecorum* LAMP primer set, as described by Jelocnik et al. [12], with the assay performed in a real-time Genie III fluorometer. The limit of detection of the *C. pecorum* LAMP assay was again evaluated using ten-fold serial-diluted, purified, and quantified *C. pecorum* E58 gDNA as the template. In our study, and comparable to Jelocnik et al. [12], the limit of detection was equivalent to 10 genome copies/µL template, with 5/5 replicates (100%) achieving positive amplification (Table S1). We also observed positive amplification at single copy dilutions; however, only in 3/5 (60%) replicates (Table S1).

### 2.2. C. pecorum Detection in Rapidly Processed Clinical Samples Using LAMP Assays

In this study, we used a total of 46 rapidly processed aqueous swab suspensions and 14 tissue lysates for isothermal *C. pecorum* testing (Table S2). Swabs were rapidly processed in water, followed by vortexing and heat lysis, while for the tissues we used the commercially available One-Step DNA Extraction G-Xtract solution (Adelaide, SA, Australia) (Figure S1). Following isothermal testing, DNA was extracted from rapidly processed samples and tested using in-house reference *C. pecorum* qPCR assay, followed by a comparison of isothermal and qPCR results (Figure S2). 

### 2.3. C. pecorum LAMP Using Swab Suspensions

Isothermal testing of rapidly processed swab suspension samples revealed a moderate agreement with the reference in-house *C. pecorum* qPCR assay, as indicated by Kappa of 0.592 and an 80.40% overall agreement (Table 1 and Table S2). Discrepant results were observed for nine swab samples where they were negative by LAMP but positive by qPCR (Table S2). Following DNA extraction and qPCR testing, seven of these samples had a geometric mean of 29.44 genome copies/µL of DNA, while sample 8 had 301.01 genome copies/µL of DNA. The last discrepant sample (38) was detected at 29 min and 15 s and had recorded melt; however, this was deemed as negative due to amplification time being below the agreed detection limit (Table S1).

### 2.4. C. pecorum LAMP Using Tissue Lysates

Isothermal testing of 14 rapidly processed tissue samples revealed a substantial agreement with the reference in-house *C. pecorum* qPCR assay, as indicated by Kappa of 0.696 and an 85.71% overall agreement (Table 1 and Table S2). Discrepant results were observed for only two tissue samples (55 and 57), with a geometric mean of 21.42 genome copies/µL of DNA, as determined by qPCR. 

### 2.5. C. pecorum LAMP Testing of Paired Swab and Tissue Samples

There was a substantial agreement between the *C. pecorum* LAMP when testing lysed tissue and rapidly processed swabs, as indicated by Kappa of 0.65 and an overall agreement of 85.71% using 14 paired samples (Table 2). Two sets of paired samples (8 swab and 50 tissue, and 36 swab and 55 tissue) recorded discrepant results (Table S2). Sample pair 8 swab and 50 tissue had a positive LAMP result for the tissue only, and a positive qPCR result for both. As above, sample 8 swab had 301.1 genome copies/µL DNA, while sample 50 tissue had 368.2 genome copies/µL DNA, respectively (Table S2). For the discrepant results for sample pair 36 swab and 55 tissue, swab 36 tested positive by LAMP, but not the paired tissue sample 55. As above, the tissue sample 55, testing negative by LAMP, also conflicted with the qPCR result. 

### 2.6. Overall C. pecorum LAMP and qPCR Agreement

**Figure 1:**
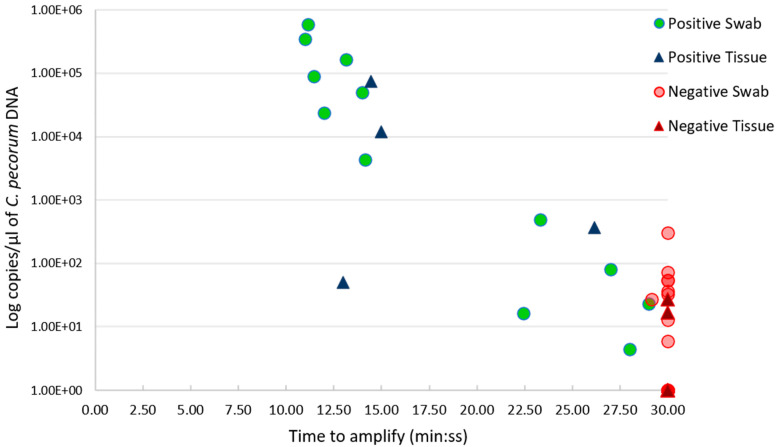
Scatter plot showing the relationship between the *C. pecorum* genome copies/µL of extracted DNA (*Y* axis), and LAMP results, presented as time to amplify (min: ss) (*X* axis). Positive LAMP results are outlined in green circles for swab samples, and in dark blue triangles for tissue samples. Negative LAMP results are denoted by red circles for swab samples, and dark red triangle for tissue samples.

In total, we had an overall agreement of 81.67% (49/60 samples; 74.42% PA; 85.71% NA) between the two tests. Of those, 16/60 samples (12 swabs and four tissues) were positively congruent, and 33/60 samples (25 swab and eight tissue) were negatively congruent between LAMP and qPCR results, respectively (Table 1 and Table S2, Figure 1). For the 11 (nine swab and two tissue) samples yielding discrepant results, negative by LAMP but positive by qPCR, we observed that these results are mainly attributed to the 10 samples with low genome copy numbers, ranging from six to 71.5 genome copies/µL of extracted DNA, with the geometric mean of 27.39 copies/µL of DNA (Figure 1, Table S2). Only the discrepant swab sample 8, negative by LAMP but positive by qPCR with a 301.01 genome copy number/µL of DNA, was outside this range. If this outlier sample is included, the discrepancy ranges from six to 301.01 genome copies/µL of extracted DNA, with the geometric mean of 34.06 copies/µL of DNA (Figure 1, Table S2).

### 2.7. Preliminary Evaluation of the Use of LAMP Assay and the Real-Time Fluorometer as a POC Diagnostic Tool

Isothermal testing, including sample processing, primer mix, and reaction preparation; use of the Genie III Fluorometer; and interpretation of results was completed at the University of the Sunshine Coast by the authors, including a practicing veterinarian with no prior molecular experience and a research veterinarian with previous LAMP and Genie III experience. Briefly, the veterinarian with no prior molecular experience received training for one day (which included all of the above) and performed rapid swab processing on over half of the samples (Table S2) and six LAMP assays with minimal assistance from experienced team members. On average, the veterinarian with no prior molecular experience took 90 min from rapid swab processing to complete the LAMP assay which included six samples plus a negative and positive control per run. 

## 3. Discussion

This pilot study has demonstrated the successful use of rapidly processed swab and tissue samples to detect livestock *C. pecorum* infections using LAMP, presenting an additional proof of concept that rapid isothermal diagnostics can be applied at the POC or clinical setting using sheep samples. The LAMP assays that were run in the Genie III fluorometer were easily and quickly performed, and interpretation of results were straightforward for a veterinarian without molecular experience, with positive results being easily characterizable by amplification time and specific melt curves. 

In practice, detection of *C. pecorum* requires sending samples to a specialised veterinary laboratory where nucleic acid testing, such as qPCR, can be performed. It is evident from this pilot study and others [14,16,17] that LAMP has the potential to be applied as a rapid, POC diagnostic tool for veterinarians performing various disease investigations in livestock species. This has numerous benefits in a veterinary setting including the ability to quickly provide results to farmers and direct disease management strategies in shorter timeframes, and with lower cost compared to other molecular methods. Rapid POC testing allows practitioners to select the most appropriate tissue samples to submit for further testing to confirm presence or absence of infectious agent (e.g., microbial culture or molecular diagnostics) and associated pathology (e.g., immunohistochemistry). The rapid sample processing methods successfully demonstrated here could be extrapolated to other important livestock pathogens, including the closely related *C. abortus* which is exotic to Australia.

Overall, there was good congruence between LAMP and the reference qPCR, averaging an 83.1% agreement between the two tests, especially when testing higher load samples (≥100 copies/µL). However, the sensitivity of LAMP appears to be reduced when testing low load samples, as most discrepant results were noted in swab samples where DNA copy number was in a range of six to 71.5 genome copies/µL of extracted DNA. This may not be a concern for disease investigations, as subclinical shedding and infected mucosal sites with low bacterial DNA copy numbers are less likely to be associated with disease [1,18]. However, improved sensitivity is required to reliably rule out *C. pecorum,* especially in low load samples and in cases with negative LAMP results, and qPCR assays should be performed to confirm the absence of *C. pecorum* DNA. It may be possible to improve the sensitivity of this LAMP assay by processing swab samples with G-Xtract solution (Geneworks, Australia) or fast polymerase mixes due to their superior ability to lyse cells via proteolysis compared to the rapid aqueous swab processing method used here. However, the benefits of these alternative methods of rapid swab processing prior to LAMP assaying requires further investigation. 

Besides the rapid sample processing by vortexing and heat lysis, we also evaluated the use of commercially available rapid DNA extraction buffer G-Xtract (Geneworks, Australia), allowing for crude DNA extraction from tissue samples. Analysis of the paired swab and tissue samples revealed a high congruence of 85.71% between swab and tissue sample LAMP results. This indicates that the swab sampling of organs is a suitable method and avoids the need for collection and segmentation of tissue samples during necropsy and the long process of tissue lysis. 

There are two other important limitations of LAMP when testing samples collected during field investigations where contamination and autolysis are common, particularly for aborted material. Firstly, LAMP does not distinguish pathogenic from non-pathogenic *C. pecorum* strains. This is also a limitation of currently available *C. pecorum* qPCRs [8], and is especially a concern for aborted material, which can easily be contaminated with non-pathogenic gastrointestinal *C. pecorum* strains. Nevertheless, the benefit of using *C. pecorum* detection by LAMP with the rapid swab processing protocol described by Jelocnik et al. [12] is that residual swab suspension can be used for DNA extraction and molecular characterisation, unlike other protocols described for rapid swab processing for *C. pecorum* LAMP [13]. In addition to determining strain pathogenicity in positive samples, supportive histopathological data are recommended for a definitive diagnosis. Secondly, while LAMP assays are generally tolerant of inhibitory factors [19], there may be some cases where inhibitors could impact results, especially for aborted samples where contamination and autolysis are frequently encountered [20]. For example, liver swab sample 8 perhaps contained inhibitors as it did not amplify in LAMP but was positive on qPCR upon DNA extraction. 

Acknowledging that a limited sample size and type was used in this study, a recommendation for further investigation into the sample characteristics that impact the sensitivity, reliability, and robustness of results is required. This will involve testing with a larger number and variety of samples to determine the impact of swab type (organs/tissues, rectal, vaginal, conjunctival, synovial, preputial, and other), clinical manifestation of infection (conjunctivitis, polyarthritis, abortion, other; symptomatic vs. asymptomatic) and sample and swab storage. Jelocnik et al. [12] has previously demonstrated that this *C. pecorum* LAMP assay is species-specific using a limited catalogue of DNA extracted from related chlamydial species (*Chlamydia psittaci, Chlamydia pneumoniae, Chalmydia abortus, Chlamydia suis, Chlamydia trachomatis, Chlamydia murridarum, Chlamydia caviae*) and other bacterial organisms. Further species specificity using expanded bacterial, viral, and protozoal samples is needed, as a myriad of taxa is known to infect sheep [20].

Due to the importance of ruling out exotic agents in abortion investigations for Australia’s trade status [20], an opportunity exists for cost-effective screening of larger numbers of samples by LAMP assays to provide comprehensive data to demonstrate freedom from disease. The development of multi-species (or a panel of specific pathogens) microfluidic chip testing capabilities has been demonstrated for the rapid isothermal detection of equine respiratory pathogens [21]. The development of an ‘abortion panel’ that contains common endemic agents and exotic pathogens would provide additional benefits for the livestock industry where numerous endemic and exotic agents can be screened simultaneously to guide further diagnostic workup and provide early notification of the zoonotic potential of the abortion outbreak. 

We have successfully demonstrated the application of LAMP using rapidly processed swabs and a commercial tissue lysis kit for rapid detection of *C. pecorum* in ovine tissues. The substantial congruence with in-house reference qPCR and simplicity of the LAMP protocol using minimally processed samples means that this assay could easily be employed in a laboratory setting as a rapid screening method or as part of a veterinary clinic’s in-house testing. However, it is evident that the *C. pecorum* LAMP assay sensitivity is poor for some samples and, because of this, samples with negative LAMP results should be cross-checked with qPCR assays.

## 4. Materials and Methods

### 4.1. Samples

This study used retrospective frozen tissue samples from ovine abortion and stillbirth investigations that were collected between 2018 and 2019 as part of a previous study where *C. pecorum* was detected [8] and was approved by Murdoch University Animal Ethics Committee (R3004/17). The testing and use of these tissues were approved by University of the Sunshine Coast Animal Ethics approval exemption (ANE2057). From 29 tissue samples, a total of 40 swabs from cotyledon (*n* = 16), foetal liver (*n* = 22), and foetal lung (*n* = 2) were obtained using individually packed Minitip Rayon dry Aluminium shaft swab (Copan, Brescia, Italy) (Table S2). An additional six vaginal swabs that were opportunistically collected in the study by Clune et al. [8] were also used. Out of the 29 available tissues, six cotyledon and eight liver tissue samples were also selected to evaluate rapid testing of tissues using G-Xtract solution (Geneworks, Adelaide, SA, Australia) (Table S2, Figure S1). The general workflow from this study is outlined in Figure S2. 

### 4.2. Swab and Tissue Processing

Swabs were rapidly processed in 300 µL of water by vortexing and heat lysis at 90 °C for 10 min, as previously described [11] (Figure S1). 

To achieve rapid lysis of tissue samples (and DNA release from the cells), we used the commercially available One-Step DNA Extraction G-Xtract solution (Geneworks, Adelaide, SA, Australia). Small pieces of tissues (3–5 mm × 3–5 mm) were excised with scalpel blades or tissue scissors and placed in an Eppendorf tube containing 300 µL of the G-Xtract solution (Geneworks, Adelaide, SA, Australia) (Figure S1). The tube was vortexed for 15 s and incubated on 65 °C for 20 min with occasional vortexing. Following the incubation, the samples were then heat lysed on 98 °C for 2 min. Prior to LAMP testing, the swab suspensions and tissue lysates were briefly centrifuged and cooled to room temperature.

### 4.3. C. pecorum Isothermal Assays

In this study, we used the *C. pecorum* LAMP primer set targeting 209 bp of the conserved *C. pecorum* hypothetical protein gene from Jelocnik et al. [12]. All LAMP assays in this study were performed in 25-µL reaction volumes, consisting of 15 µL of isothermal master mix ISO001 (Optigene, Horsham, UK), 5 µL of primers mix (at 0.2 µM F3 and B3, 0.8 µM FIP and BIP, and 0.4 µM LF and LB), and 5 µL of template,. This was then run at 65 °C for 30 min, followed by a denaturation step of 98–80 °C at a rate of 0.05 °C/s in the Genie III real-time fluorometer (Optigene, Horsham, UK) to create a high-resolution melt curve. Positive (cultured *C. pecorum* E58 DNA) and negative (MilliQ water) controls were included in each assay. A sample was deemed positive if *C. pecorum* DNA was detected within ≤29 min and had a high-resolution melt (HRM) of 83.5 ± 1 °C. 

The limit of detection using *C. pecorum* isothermal assay was evaluated using 1 µL of quantified *C. pecorum* E58 DNA in serial dilutions from 10^3^ (tested in triplicate) to 10^−1^ copies/µL (tested as five replicates; Table S1). 

In order to perform a comparison to the reference in-house *C. pecorum* qPCR assay [2], the remaining 250 µL of the swab suspension and tissue lysates was used for DNA extraction using QiaAMP DNA mini kit, as per manufacturer instructions (Qiagen, Chadstone, Vic, Australia). The in-house qPCR assays for *C. pecroum* targeted 209 bp amplicon generated by F3 and B3 primers. Briefly, all qPCR assays were carried out in a 15 µL total volume, consisting of 7.5 µL of iTaq master mix (Biorad, Gladesville, NSW, Australia), 0.5 µL of each 10 µM forward and reverse primer (Sigma Aldrich, Castle Hill, Australia), 3.5 µL of MiliQ water, and 3 µL of DNA template. The qPCR assays were run for 35 cycles, and in each qPCR assay positive (cultured *C. pecorum* E58 DNA) and negative (mix only and MiliQ water) controls were included. Each sample was tested in duplicate, and in this study, a sample was deemed positive if *C. pecorum* DNA detected in duplicate had a Cq value of <33.5 and high-resolution melt (HRM) of 77.5 °C ± 0.5 °C. The limit of detection and *C. pecorum* genome copy number in samples (tested in duplicate) was quantified by plotting the crossing points against a standard curve generated from triplicates of the ten-fold serial dilution of 10^6^ to 10^0^ copies/µL of previously quantified *C. pecorum* DNA. Samples with a discrepant result between LAMP and qPCR testing were retested to confirm the results. 

### 4.4. Testing of Spiked Samples

After all isothermal testing of samples, we performed testing of spiked samples. A 1 cm × 1 cm portion of the originally tested negative tissues (sample 23 cotyledon, sample 37 cotyledon and sample 39 liver) were “single-blind” spiked with various concentrations of chlamydial elementary bodies. An aliquot of a 500-µL *C. pecorum* strain E58 elementary bodies (EBs) in sucrose phosphate glutamate (SPG) was defrosted. Previously, EBs concentration was estimated to be 10^8^ EBs/mL (10^5^ EBs/µL) by microscopy. For swab samples, 40 (cotyledon), 46 (cotyledon) and 41 (liver), we added 20 µL, 15 µL, and 10 µL of the EBs, respectively, to the swabs in 300 µL of water. Swabs were then processed as above. For tissue samples 59 (cotyledon) and 60 (liver), a small tissue fragment was excised and placed in a G-Xtract buffer, followed by addition 10 µL of EBs into each tube. Tissue samples were then vortexed and placed on heat for lysis, as described above. 

These “spiked” tissues were then treated as unknown samples by the operator and processed as swabs and/or tissues, as outlined above. The spiked samples were denoted as “new” swab samples 40, 41, and 46; and “new” tissue samples 59 and 60. The samples swab 40 and tissue 59, and swab 41 and tissue 60 were paired. The isothermal testing was performed as above, followed by DNA extraction and qPCR testing, also outlined above. Their results were included in calculations, as the operator performing the testing was unaware that these were spiked. This information was revealed upon completion of all analyses in this study. 

### 4.5. Test Congruence and Statistical Analyses

The results for (i) rapidly processed swabs and tissue lysates tested with LAMP, and DNA extracts of the same samples tested with in-house qPCR and (ii) rapidly paired and processed swabs and tissue lysates tested with LAMP were compared by calculating Kappa, positive and negative agreement proportions, and McNemar’s Chi-squared value with a 95% confidence using the online diagnostic test evaluation modules (https://epitools.ausvet.com.au/comparetwotests; accessed on June–July 2021). It is suggested the Kappa value be interpreted as follows: values ≤0 as indicating no agreement, 0.01–0.20 as none to slight, 0.21–0.40 as fair, 0.41–0.60 as moderate, 0.61–0.80 as substantial, and 0.81–1.00 as almost perfect agreement.

## Figures and Tables

**Table 1:** Comparison of *C. pecorum* LAMP assays using rapidly processed swab and tissue samples to in-house reference *C. pecorum* qPCR.

*C. pecorum* LAMP testing swabs	**Reference *C. pecorum* qPCR with Swabs**			
		Positive	Negative	Total
	Positive	12	0	12
	Negative	9	25	34
	Total	21	25	46
	Kappa (95% CI; *p*-value)	0.592 (0.3733–0.8101; 0.00)		
	McNemar’s Chi square (*p*-value)	7.111 (0.008)		
	Overall agreement	80.4% (72.73% PA; 84.75%NA)		
*C. pecorum* LAMP testing tissue lysates	**Reference *C. pecorum* qPCR with Lysed Tissue**			
		Positive	Negative	Total
	Positive	4	0	4
	Negative	2	8	10
	Total	6	8	14
	Kappa (95% CI; *p*-value)	0.696 (0.3237–1.0676; 0.0031)		
	McNemar’s Chi square (*p*-value)	0.5 (0.480)		
	Overall agreement	85.71% (80% PA; 88.89% NA)		

PA: positive agreement, NA: negative agreement.

**Table 2:** Comparison of *C. pecorum* LAMP assays using paired rapidly processed swabs and lysed tissue samples.

*C. pecorum* LAMP with lysed tissue	***C. pecorum* LAMP with Swabs**			
		Positive	Negative	Total
	Positive	3	1	4
	Negative	1	9	10
	Total	4	10	14
	Kappa (95% CI; *p*-value)	0.65 (0.2068–1.0932; 0.0075)		
	McNemar’s Chi square (*p*-value)	0.5 (0.480)		
	Overall agreement	85.71% (75% PA; 90% NA)		

PA: positive agreement, NA: negative agreement.

## Data Availability

Not applicable.

## Supplementary Materials

The following are available online at https://www.mdpi.com/article/10.3390/pathogens10091157/s1, Table S1: Limit of detection of *C. pecorum* LAMP assay in this study. Table S2. Clinical samples used for isothermal and qPCR testing in this study. Figure S1: Overview of the workflow for isothermal LAMP testing and comparisons to qPCR assays from this study. Figure S2: Swab/tissue processing and isothermal assay performed in Genie III fluorometer. A: Ovine tissues and swabs taken from the collected tissues; B: Tissue in G-Xtract solution and swabs in water; C: Samples after vortexing and heat lysis; D: Isothermal assay components: isothermal mix primer mix and water; E: Mix dispensing into Genie III tubes; and F: Genie III screen outputs with amplification curves on top screen and melt on the bottom screen.
