# Supplementary material for: Real-Time Fluorometric Isothermal LAMP Assay for Detection of Chlamydia pecorum in Rapidly Processed Ovine Abortion Samples: A Veterinary Practitioner’s Perspective
Source: Pathogens. 2021 Sep 8;10(9):1157. doi: 10.3390/pathogens10091157 (PMC8470028; doi:10.3390/pathogens10091157)
Supplement: Supplementary file 1 [file pathogens-10-01157-s001.zip › Table S1.pdf]

Table S1. Limit of detection of *C. pecorum* LAMP assay in this study.

Dilution (copies/ µl DNA template)	Time to amplify (min:ss)	Melt (°C)	Mean time (SD)	Melt (SD)
1000	12:00	84.42		
1000	12:15	84.37	12.00 (0.25)	84.42 (0.05)
1000	11:45	84.46		
100	13:30	84.36		
100	14:00	84.37		
100	15:15	84.37	14.00 (0.95)	84.34 (0.04)
100	14:30	84.32		
100	12:45	84.27		
10	18:30	84.42		
10	18:15	84.42		
10	20:00	84.52	19:48 (1.69)	84.38 (0.11)
10	19:45	84.29		
10	22:30	84.24		
1	24:15	83.45		
1	NA	NA		
1	26:30	83.50	NA	NA
1	29:15	83.54		
1	NA	NA		
0.1	NA	NA		
0.1	NA	NA		
0.1	NA	NA	NA	NA
0.1	NA	NA		
0.1	NA	NA		

SD: standard deviation; NA: no amplification.
