# Supplementary material for: Real-Time Fluorometric Isothermal LAMP Assay for Detection of Chlamydia pecorum in Rapidly Processed Ovine Abortion Samples: A Veterinary Practitioner’s Perspective
Source: Pathogens. 2021 Sep 8;10(9):1157. doi: 10.3390/pathogens10091157 (PMC8470028; doi:10.3390/pathogens10091157)
Supplement: Supplementary file 1 [file pathogens-10-01157-s001.zip › Table S2 revised.pdf]

Table S2: Clinical samples used for isothermal and qPCR testing in this study.

Sample	Anatomical tissue	Sample type	LAMP result[1]	qPCR result for DNA extract[2]	Average CT (SD)	High Resolution Melt (^{0}C)	Average copy number/µl of DNA
1	Liver	Swab	NEG	NEG			
2	Liver	Swab	NEG	NEG			
3[A]	Liver	Swab	NEG	NEG			
4	Liver	Swab	POS (27.00, 84.24)	POS	29.09 (0.71)	78	79.95
5[B]	Cotyledon	Swab	NEG	NEG			
6[C]	Cotyledon	Swab	POS (14.00, 84.04)	POS	19.87 (0.08)	77.75	49746.33
7	Cotyledon	Swab	POS (22.45, 84.17)	POS	31.36 (0.0)	77.5	16.40
8[D]	Liver	Swab	NEG	POS	27.19 (0.09)	78	301.01
9	Liver	Swab	NEG	NEG			
10[E]	Liver	Swab	NEG	POS	32.83 (0.10)	78	6.00
11	Cotyledon	Swab	NEG	NEG			
12	Cotyledon	Swab	NEG	POS	29.65 (0.09)	78	54.00
13	Liver	Swab	NEG	NEG			
14	Lung	Swab	NEG	NEG			
15	Lung	Swab	NEG	NEG			
16	Liver	Swab	NEG	POS	29.3 (0.13)	78	54.09
17	Liver	Swab	NEG	NEG			
18	Cotyledon	Swab	NEG	POS	29.25 (0.46)	78	71.51
19	Liver	Swab	NEG	NEG			
20	Liver	Swab	NEG	NEG			
21[F]	Cotyledon	Swab	NEG	NEG			
22	Liver	Swab	NEG	NEG			
23	Cotyledon	Swab	NEG	NEG			
24	Liver	Swab	NEG	NEG			
25	Liver	Swab	NEG	NEG			
26	Liver	Swab	NEG	NEG			
27	Liver	Swab	NEG	NEG			
28[G]	Liver	Swab	NEG	NEG			
29[H]	Cotyledon	Swab	NEG	NEG			

30	Vaginal	Swab	NEG	NEG	33.52 (0.32)	78.5	3.71
31	Vaginal	Swab	POS (23.30, 84.48)	POS	26.48 (0.21)	77.5	494.00
32	Vaginal	Swab	POS (11.00, 84.53)	POS	17.9 (0.13)	77.5	346087.01
33	Vaginal	Swab	NEG	POS	31.7 (0.78)	77.25	12.94
34	Vaginal	Swab	POS (28.00, 84.93)	NEG	33.25 (0.73)	78	4.39
35	Vaginal	Swab	POS (11.15, 84.83)	POS	16.32 (0.07)	77.5	592268.50
36[I]	Liver	Swab	POS (29.00, 83.2)	POS	30.86 (0.34)	77.5	23.25
37[J]	Cotyledon	Swab	NEG	NEG			
38[K]	Cotyledon	Swab	BDL (29.15, 83.65)	POS	30.67 (0.12)	78	27.00
39[L]	Liver	Swab	NEG	NEG			
40[M]*	Cotyledon	Swab	POS (12.00, 84.75)	POS	20.92 (0.16)	77.5	23909.98
41[N]*	Liver	Swab	POS (14.15, 83.94)	POS	23.38 (0.52)	77.5	4296.60
42	Cotyledon	Swab	POS (11.45, 83.48)	POS	19.04 (0.3)	77	88772.04
43	Cotyledon	Swab	NEG	POS	30.24 (0.43)	78	35.84
44	Cotyledon	Swab	NEG	POS	30.34 (0.59)	77.5	33.00
45	Liver	Swab	NEG	NEG			
46*	Cotyledon	Swab	POS (13.15, 83.34)	POS	18.5 (0.15)	77.5	165185.98
47[A]	Liver	Tissue lysate	NEG	NEG			
48[B]	Cotyledon	Tissue lysate	NEG	NEG			
49[C]	Cotyledon	Tissue lysate	POS (14.45, 83.69)	POS	19.29 (0.04)	78	74562.19
50[D]	Liver	Tissue lysate	POS (26.15, 83.07)	POS	26.9 (0.02)	78	368.52
51[E]	Liver	Tissue lysate	NEG	NEG			
52[F]	Cotyledon	Tissue lysate	NEG	NEG			
53[G]	Liver	Tissue lysate	NEG	NEG			
54[H]	Cotyledon	Tissue lysate	NEG	NEG			
55[I]	Liver	Tissue lysate	NEG	POS	30.63 (0.00)	78	27.00
56[J]	Cotyledon	Tissue lysate	NEG	NEG			
57[K]	Liver	Tissue lysate	NEG	POS	31.3 (0.06)	78	17.00
58[L]	Liver	Tissue lysate	NEG	NEG			
59[M]*	Cotyledon	Tissue lysate	POS (15.00, 83.08)	POS	21.91 (0.07)	77.5	11983.38
60[N]*	Liver	Tissue lysate	POS (13.00, 83.07)	POS	29.77 (0.81)	78	49.75

[A-N] Samples with the same superscript are paired samples.

[1]LAMP results are expressed: time to amplify (min), melt (^0C). Samples with no amplification, and/or amplification time of > 29 min and melt below threshold (below detection level) are considered NEGATIVE.

[2]Samples with no amplification, and/or Cq of > 33.5 and melt below threshold are considered NEGATIVE.

*Spiked samples.

POS: positive; NEG: negative
