# Supplementary figures and images for: Real-Time Fluorometric Isothermal LAMP Assay for Detection of Chlamydia pecorum in Rapidly Processed Ovine Abortion Samples: A Veterinary Practitioner’s Perspective
Source: Pathogens. 2021 Sep 8;10(9):1157. doi: 10.3390/pathogens10091157 (PMC8470028; doi:10.3390/pathogens10091157)

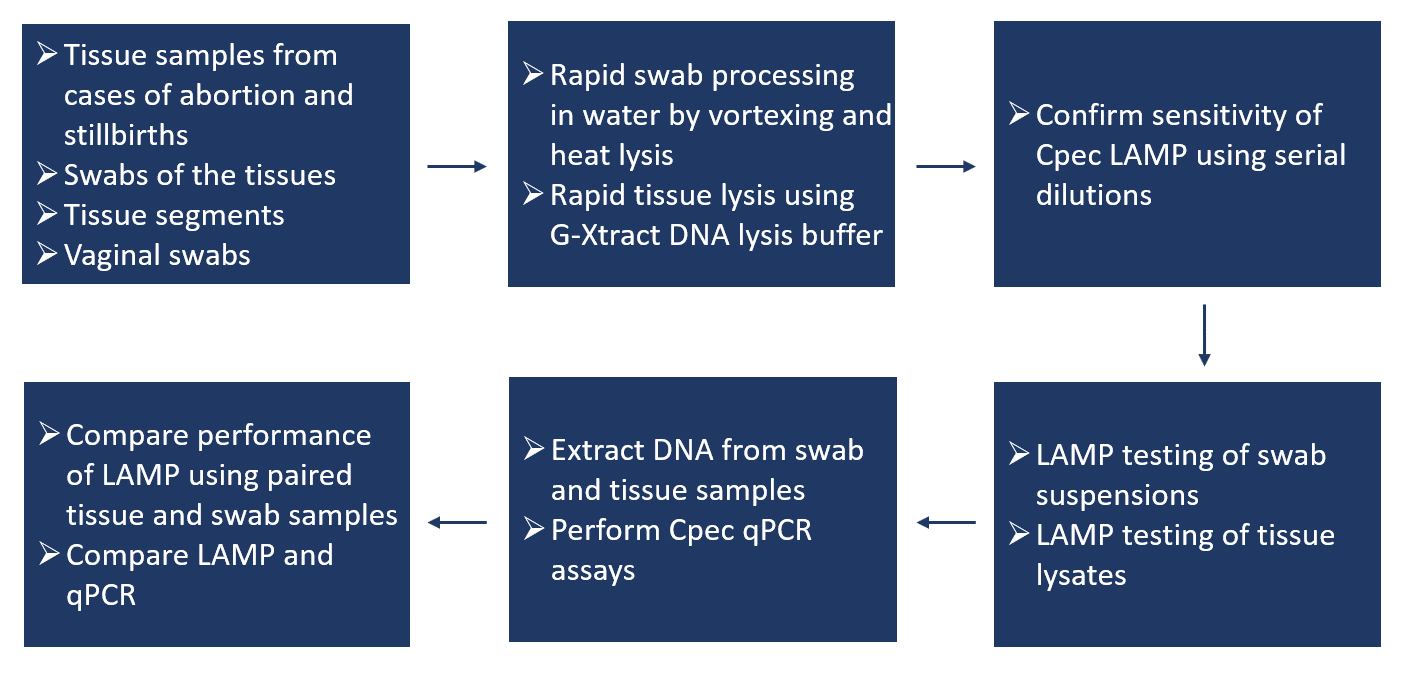

Supplement: Supplementary file 1 [file pathogens-10-01157-s001.zip › Figure S1_workflow.png]

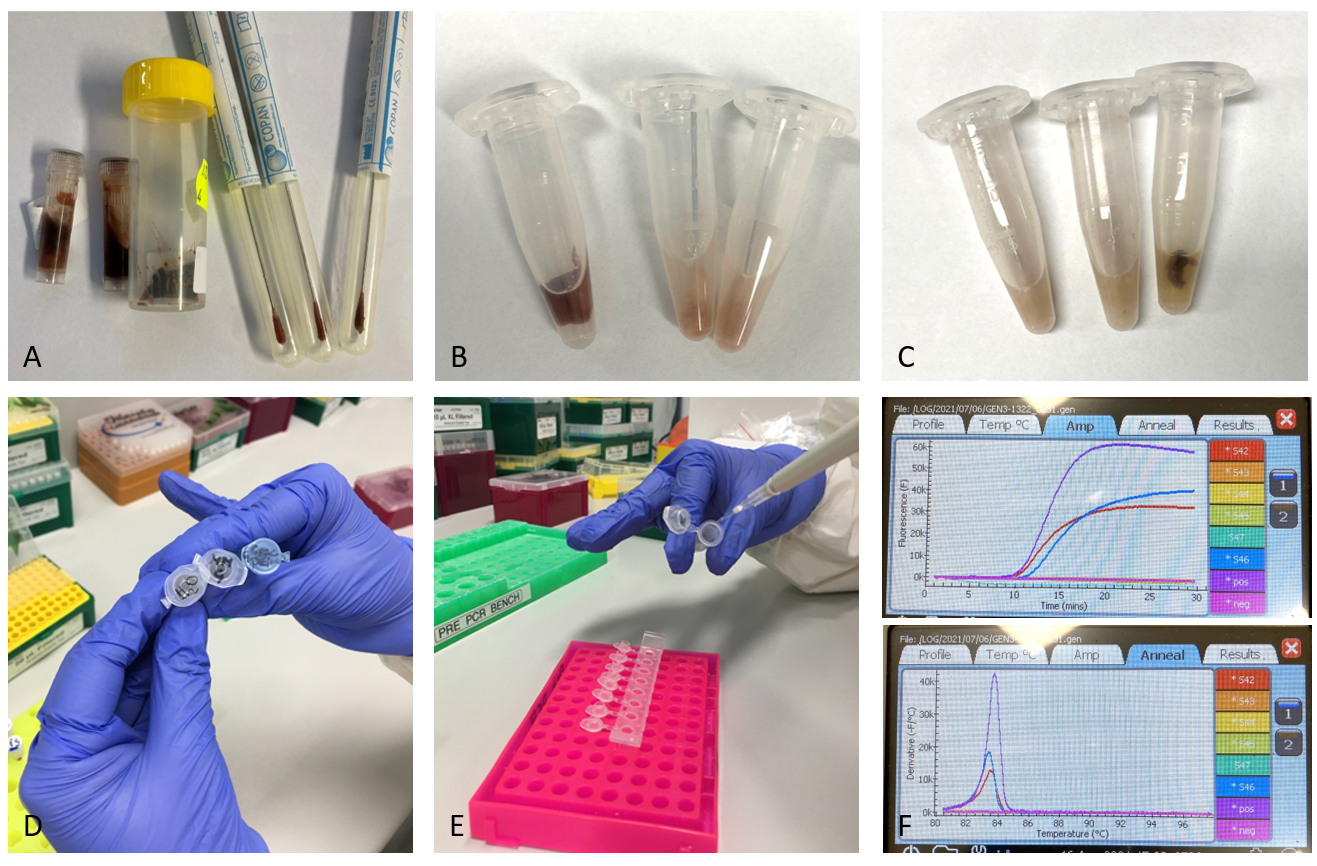

Supplement: Supplementary file 1 [file pathogens-10-01157-s001.zip › Figure S2_swabs.png]
